# Key Resting Echocardiographic Parameters for the Estimation of Exercise Parameters of Peak VO2, Heart Rate Recovery, and Ventilatory Efficiency

**DOI:** 10.3390/jcm14093013

**Published:** 2025-04-27

**Authors:** Kalyan Chaliki, Arundhati Sharma, Anubhuti Sharma, Claire Yee, Hari Chaliki, Satyajit Reddy

**Affiliations:** 1 Division of Cardiology, The University of Arizona College of Medicine—Phoenix, Phoenix, AZ 85004, USA; kalyanchaliki@gmail.com; 2 Department of Cardiovascular Diseases, Mayo Clinic, Scottsdale, AZ 85054, USA; arundhati.fnu@mayo.edu (A.S.); anubhuti.fnu@mayo.edu (A.S.); yee.claire@mayo.edu (C.Y.); chaliki.hari@mayo.edu (H.C.)

**Keywords:** cardiopulmonary exercise test, CPET, Echo, VO2 max

## Abstract

**Background/Objectives**: The peak oxygen consumption (VO2) during cardiopulmonary exercise testing (CPET) is a strong predictor of all-cause mortality. The cardiac output, a key determinant of VO2, can be assessed using resting echocardiographic parameters. The heart rate recovery and ventilatory efficiency (VE/VCO2 slope) from CPET offer additional insights into cardiovascular fitness. **Methods**: This study aimed to identify resting echocardiographic parameters that predict the percentage of predicted peak VO2, heart rate recovery, and VE/VCO2 slope in a general cardiology population. This retrospective analysis included 1909 patients who underwent echocardiography within 3 months of CPET from 2017 to 2022. Patients with potentially confounding co-morbid conditions were removed. Spearman correlations were used to compare 19 echocardiographic parameters to peak VO2, heart rate recovery, and the VE/VCO2 slope, followed by multiple linear regression of peak VO2. **Results**: Eleven echocardiographic parameters correlated with peak VO2, with the strongest correlations seen with the left ventricular stroke volume index (R = 0.284, *p* < 0.001), mitral valve medial annular a’ wave velocity (R = 0.142, *p* < 0.0001), and mitral E-to-e’ ratio (R = −0.117, *p* < 0.0001). The left ventricular diastolic parameters and mitral E/A ratio correlated strongly with the heart rate recovery and VE/VCO2 slope. The multiple linear regression analysis identified the left ventricular mass index, stroke volume index, mitral valve E wave velocity, tricuspid valve regurgitation peak systolic velocity, tricuspid lateral annular systolic velocity S’, and left atrial volume index as independent predictors of peak VO2 (R^2^ = 0.191). **Conclusions**: The left ventricular stroke volume, diastolic function, and RV systolic function markers are significant predictors of cardiopulmonary fitness, aiding clinical decision-making in patients without CPET data.

## 1. Introduction

The peak rate of oxygen volume used during aerobic exercise (peak VO2) is a strong predictor of all-cause mortality [1,2], cardiovascular mortality [3], and cardiac morbidity [4]. Cardiopulmonary exercise testing (CPET) is a common way to evaluate peak VO2 in adults. While peak VO2 is a complex interaction of multiple physiologic interactions, the cardiac output is the most common limiter of peak VO2 in healthy adults [5,6]. The cardiac output, the product of stroke volume and heart rate, is achieved through a combination of several aspects of heart function that can be assessed via resting echocardiography. While CPET provides the gold standard evaluation of functional capacity, CPET equipment and personnel may not be available in all healthcare centers, and patients may not be able to perform a stress test due to neurological or muscular issues. In contrast, resting echocardiography is a routine test with widespread use [7]. Although certainly resting echocardiography cannot replace CPET, there may be value in understanding the relationship between these two diagnostic modalities. Little is known, however, about the relationship between using multiple resting echocardiographic parameters and peak VO2, outside of specific subpopulations such as heart failure patients [8,9,10,11,12] and elite athletes [13,14,15,16]. Broader general cardiology populations, in contrast, provide a unique clinical diagnostic challenge in which the definitive relation between functional capacity and echocardiographic variables often unclear.

CPET can also provide value through information on heart rate recovery and ventilatory efficiency (VE/VCO2 slope). Heart rate recovery, the reduction in heart rate immediately after maximal effort exercise, typically measured at 1 or 2 min, is a proposed surrogate marker of autonomic health. Faster heart rate recovery is considered a favorable indicator of parasympathetic health and is an independent predictor of mortality in both heart failure patients [17] and coronary artery disease patients [18]. A study of 282 patients with coronary artery disease found that an abnormal heart rate recovery (defined as less than 13 beats/min reduction after 1 min) was associated with a 2.16-fold increased risk of all-cause mortality than those without, when adjusting for age, gender, and low peak VO2 [19]. The VE/VCO2 slope is the rate of change of the increase in minute ventilation (VE) in response to an increased carbon dioxide production (VCO2) from the start of exercise to max effort. A lower VE/VCO2 slope is a sign that the body is more efficiently able to ventilate carbon dioxide out from the body and is related to mortality and prognosis in heart failure patients [20,21], as well as cardiac event rates in general cardiac patients [22]. Both heart rate recovery and the VE/VCO2 slope are important measurements to provide a more comprehensive picture of cardiovascular fitness. However, as above, prior studies assessing the relationship between recovery heart rate or the VE/VCO2 slope and resting echocardiographic parameters focused on narrow subpopulations [23,24,25,26].

In a general cardiology patient population, we sought to identify which resting echocardiographic parameters are most associated and predictive of peak VO2 (adjusted for age, sex, and body size), and which are most associated with heart rate recovery and ventilatory efficiency.

## 2. Materials and Methods

Patients aged 18 years and older were identified in the Mayo Clinic database if they had a resting transthoracic echocardiogram exam within 3 months before or after a maximum effort treadmill CPET exam, defined as reaching a Respiratory Exchange Ratio of at least 1.15, from 1 January 2017 to 31 December 2022. The CPET protocols were individualized based on each patient’s baseline functional capacity and anticipated exercise tolerance. The following standardized protocols were used: Bruce, modified Bruce, Naughton, modified Naughton, Mayo, and occasionally other patient-specific protocols (see Supplementary Materials Figure S1 for details of the protocols). A cycle ergometer and other exercise modalities were not included in this study. The protocol selection was at the discretion of the supervising clinical physiologist and physician to ensure patient safety and optimal test results. Standardized comprehensive resting TTE measurements were performed at the Mayo Clinic Arizona following the American Society of Echocardiography guidelines [27,28]. Standard 2D and Doppler measurements were obtained in the left lateral decubitus position, including left ventricular (LV) volumes, stroke volume, ejection fraction, wall thickness, and left atrial (LA) volume, using the biplane method of disks.

Mitral inflow velocities (E and A waves, E-wave deceleration time) were recorded using pulsed-wave Doppler testing with the sample volume at the mitral leaflet tips. The tricuspid regurgitation velocity was assessed via continuous-wave Doppler testing to estimate the RV systolic pressure.

Tissue Doppler imaging was used to measure the e’, a’, and S’ velocities by placing a small sample volume at the septal and lateral mitral annulus and lateral tricuspid annulus. The Nyquist settings and filters were adjusted to optimize the myocardial signal quality. All measurements were performed by experienced sonographers and interpreted by board-certified echocardiographers.

Patients were excluded if they had a history of coronary artery bypass grafting, congenital heart disease, valvular repair or replacement, severe valvular stenosis or regurgitation, a pacemaker, atrial fibrillation or flutter, pulmonary hypertension (based on right ventricular systolic pressure >40), or a left ventricular ejection fraction <50%. Echocardiographic parameters from the American Society of Echocardiography were used to exclude patients with severe aortic, mitral, pulmonic, or tricuspid stenosis or regurgitation at the time of echocardiography. A summary of the inclusion and exclusion criteria is provided in Table 1 below. The predicted values for the patients’ peak VO2 results indexed to body weight were calculated using the institutional Mayo Clinic formula, which adjusts based on age in years and sex (male predicted peak VO2 = 58.5 − age × 0.44; female predicted peak VO2 = 45.3 − age × 0.37).

The statistical analyses were conducted with SAS version 9.04. Descriptive statistics including the frequency and percentage mean, as well as the standard deviations for demographic variables of sex, age, BMI, and BSA, were calculated. Spearman correlations were then used to assess the relationships between 19 echocardiographic parameters and the percentage of predicted peak VO2 achieved, recovery heart rate, and ventilatory efficiency. Given the number of tests performed, a Bonferroni correction was used to correct for multiple testing for each set of correlations for a single outcome (e.g., one set for predicted peak VO2, one for HRR, one for the VE/VCO2 slope), with a *p*-value < 0.0025 considered to be significant. A multiple linear regression model was then built for peak VO2 including the 11 echocardiographic variables that were significantly related from the univariate analysis. The variance inflation factor (VIF) was assessed to evaluate multicollinearity among the echocardiographic variables. The VIF values ranged from 1.67 to 127.9, with higher values indicating that a given echocardiographic variable exhibited a high degree of collinearity with at least one other echocardiographic variable [29]. To address this, variables with VIF values greater than 10 were removed in an iterative manner. At each step, the variable with the highest VIF and the lowest correlation with the percentage of predicted peak VO_2_ (based on the univariate analysis) was excluded. The model was then re-run, and the VIFs were re-evaluated. This process continued until all remaining variables had VIF values below 10.

Of the 11 echocardiographic variables initially included in the regression model, the MV lateral annulus E/e’ ratio was ultimately excluded due to high collinearity with other variables. Additionally, although the mitral valve (MV) lateral annulus E/e’ ratio was significantly associated with the predicted peak VO_2_ in the univariate analysis, it was not retained in the final model, as the MV medial annulus and MV average E/e’ ratios are more commonly used in clinical practice. In the final regression model, the variables were considered statistically significant at *p* < 0.05.

## 3. Results

A total of 1909 patients were included in the study. The demographic information is shown in Table 2.

**Figure 1:**
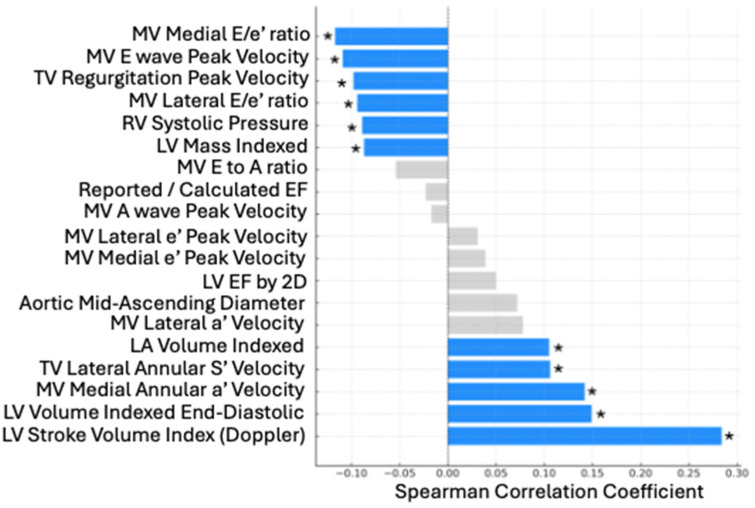
Correlations between echo parameters and percentage of predicted Peak VO2. LV, left ventricle; MV, mitral valve; TV, tricuspid valve; LA, left atrial. * Statistical significance set at *p* < 0.0025.

The Spearman correlations between the 19 echocardiographic variables and percentage of predicted peak VO2 achieved are shown in Table 3 and Figure 1. Additional descriptive statistics can be found in Supplemental Materials Table S1.

The significantly correlated variables from Table 2 were regressed against the percentage of predicted peak VO2 achieved after removing the colinear variables, yielding Table 4.

The Spearman correlations between the 19 echocardiographic variables and heart rate recovery and then separately for the VE/VCO2 slope were calculated. The same six echocardiographic variables had the highest correlations independently with heart rate recovery and the VE/VCO2 slope, and all variables were related to mitral valve parameters. These were the mitral valve A wave peak velocity, lateral annular e’ peak velocity, lateral annular E-to-e’ ratio, E-to-A ratio, medial annular e’ peak velocity, and E-to-e’ ratio. These selected results are presented in Table 5.

## 4. Discussion

This is the first study to our knowledge that assesses the association of resting echocardiographic parameters and the peak VO2, heart rate recovery, and ventilatory efficiency (VE/VCO2 slope) in a large general cardiology population. Additionally, this study is the first of its kind that attempts to predict the peak VO2 based solely on resting echocardiographic parameters.

Echocardiographic parameter correlations with peak VO2

Our study found that peak VO2 had the strongest statistically significant relationship with the LV stroke volume index, as measured separately by both the quantitative Doppler and 2D Biplane Method of Discs. This finding is similar to prior studies. Stroke volume is known to increase by up to about 40% the peak VO2 on average, after which the primary driver of cardiac output augmentation with increasing exercise intensity is heart rate elevation [30]. Our study shows that the resting stroke volume is a strong predictor of peak VO2, pointing to the idea that both baseline and exercise-induced changes in stroke volume are important in peak VO2.

Measures of cardiac chamber size, such as the left ventricle mass index, left ventricle volume index, and left atrial volume index, were significantly related with higher peak VO2. This is consistent with the Morganroth hypothesis for endurance athletes [31], and prior studies indicating heart size has a major association with peak VO2 differences between men and women [32], as well as between athletes and nonathletes [33].

Key measures of diastolic function in our study, such as the mitral valve medial E/e’ ratio, lateral E/e’ ratio, medial annular peak A wave velocity, and peak E wave velocity, were significantly correlated with VO2. However, the mitral valve medial and lateral annular e’ peak velocities were not found to be significantly correlated with VO2, despite them being more common measurements used in clinical practice to evaluate diastolic function. These results suggest that the parameters found above to be significant may be useful additional adjunctive measures to evaluate comprehensive diastolic function in the context of evaluating functional capacity. Specifically, the medial annular peak A wave velocity may be a parameter that should have more significance placed on it when evaluating functional capacity. Although the relationship between diastolic measures of heart function and peak VO2 are well established in heart failure patients [12,34] and athletes [35], this study is the first to apply it to a broader patient population not exclusively comprising heart failure patients or athletes. Although this study only included patients with normal left ventricular systolic function, a specific left ventricular ejection fraction over 50% was not significantly correlated with peak VO2. Further studies looking at all patients of all left ventricular ejection fraction ranges will be useful for the further generalizability of these results.

Additionally, there exists a well-established relationship between age and decline in diastolic function, with an increase in isovolumic relaxation time and a decrease in early diastolic mitral annular velocity [36,37], as well as an increase in late diastolic filling (A wave), leading to a decreased E/A ratio [38]. In contrast, physical stress plays an important role in improving and maintaining diastolic function. Athletes display significantly increased E/A ratios [39], with normal to increased peak early diastolic mitral annular velocities [40]; older athletes also resist age-related declines in diastolic function [41,42]. The heterogeneity of the study population, while providing generalizability, does not account for cardiac adaptations to age or patient-specific exercise volume and their interactions with CPET outcome variables. Further studies will be needed to evaluate how diastolic function, age, chronic exercise, and CPET performance are interrelated.

Certain echocardiographic parameters known from prior studies to correlate with peak VO2 were not found to have significant correlations in our data. For example, masters endurance athletes tend to have larger ascending aortic sizes [43]. However, in our sample, the mid-ascending aorta diameter was not significantly correlated with peak VO2, suggesting that dilation of the ascending aorta may require a certain threshold of physical fitness and exercise volume before it manifests.

Echocardiographic parameter correlations with heart rate recovery and ventilatory efficiency

Both the heart rate recovery and ventilatory efficiency (VE/VCO2 slope) had similar correlations with resting echocardiographic parameters, with the six highest correlated variables being the same and all related to the mitral valve function and hemodynamics. These were the mitral valve E-to-A wave ratio, average E-to-e’ ratio, lateral annular E-to-e’ ratio, A wave peak velocity, and lateral and medial annular e’ velocities. Given that a higher heart rate recovery rate but a lower VE/VCO2 slope is considered healthier, unsurprisingly, each of these echocardiographic diastolic parameters has an opposite correlation direction with each exercise parameter. For example, the E-to-A ratio and lateral and medial annular e’ velocities had a statistically significant positive correlation with heart rate recovery, while having a statistically significant negative correlation with the VE/VCO2 slope.

These diastolic function measures show strong correlations with heart rate recovery and the VE/CO2 slope, suggesting that both may reflect similar aspects of cardiovascular function, despite assessing different components of cardiorespiratory fitness. Heart rate recovery measures the parasympathetic function and autonomic homeostatic strength of an individual. On the other hand, the VE/VCO2 slope measures the ventilatory efficiency (i.e., how efficiently carbon dioxide is being removed during breathing) and incorporates pulmonary and cardiovascular function. The fact that these differing measures of cardiovascular fitness have such similar correlations with the same diastolic echocardiographic parameters is an important key finding that should be a topic of further exploration. While prior studies have linked heart rate recovery to diastolic function in a general population [44] and prior studies have linked ventilatory efficiency to diastolic function in heart failure [21,45], this study provides a unique perspective in linking both CPET variables to diastolic function in the same general cardiology population. Physiologically, a more athletic and functional heart has high compliance and early diastolic recoil of the LV, facilitating the rapid transfer of blood from the left atrium during exercise. A 2021 study found that the mitral annular dimensions and tenting volumes are significantly larger in athletes in comparison to nonathlete controls [46], which again provides support for the impact that endurance physical activity can have on the function and anatomy of the mitral valve.

Multiple linear regression of peak VO2 from echocardiographic parameters

This study is the first of its kind to attempt to predict the percentage of predicted peak VO2 achieved solely from resting echocardiographic parameters. Given differences in accessibility, expense, and professionals trained in the interpretation of CPET results, there can be value in predicting the functional capacity solely from the more widely used resting echocardiogram. However, peak VO2 is a complex interaction of cardiac, pulmonary, vascular, muscular, and mitochondrial functions. Despite these interactions, our model had an R^2^ = 0.191, indicating that 19.1% of the variability in peak VO2 in this population was explained solely by these resting echocardiographic parameters (LV mass index, stroke volume index, mitral valve E wave velocity, tricuspid valve regurgitation peak velocity, tricuspid valve lateral annular peak velocity, and left atrial volume index). The correlational directions of each of these variables with peak VO2 are physiologically consistent apart from the mitral valve E-wave velocity. This finding likely reflects a nonlinear, bimodal relationship in which elevated E-wave velocities can be observed at both extremes of physiological fitness. Younger, well-conditioned individuals may have a high E-wave velocity reflecting brisk relaxation and diastolic filling [47], while older, deconditioned patients may also have elevated E-wave velocities indicative of impaired diastolic function and elevated left atrial pressures [48]. Given that the majority of our study population consisted of older adults referred for clinical evaluation, the observed negative correlation in the regression model was likely driven by the latter group.

Further research will be needed to explore how this model can be improved with the use of additional readily available diagnostics such as electrocardiography, with new algorithms in machine learning, or within certain subpopulations. Additionally, this shows proof of concept for the continued evaluation of using echocardiography for determining measures of cardiopulmonary fitness and health prognostication.

## 5. Limitations

Our study has several limitations. First, our sample consisted of patients from a general cardiology clinic, often with an indication of dyspnea, who did not have a significant confounding co-morbid condition. Although our sample was well-distributed with age and sex, this limits generalizability of our findings to broader patient populations and introduces potential sampling bias, as our population sample was likely less physically fit than the average population, given that the mean percent-predicted peak VO2 in our study population was 81.4% (where 100% predicted peak VO2 would represent an average expected cardiorespiratory fitness level). Secondly, while our patient exclusion criteria enhanced the internal validity of the study, future research studies should explore how certain significant cardiac pathologies such as arrhythmias, severe valvular dysfunction, a history of coronary artery bypass surgery might influence the relationship between echocardiography and CPET. Additionally, recent studies have found an association between a pectus excavatum deformity and impaired cardiopulmonary function [49], which was not assessed in this study and may be a confounding demographic factor. There is also a potential for clinical or physiological changes to have occurred during the three-month interval between the echocardiography and CPET, which may have influenced the observed associations between the resting cardiac structure or function and exercise capacity. Finally, an internal institutional predictive formula was used to calculate the predicted peak VO2 based on age, sex, and body size, which may slightly differ from other normative values.

## 6. Conclusions

Our study shows that echocardiographic measures of diastolic function are highly correlated with peak VO2, heart rate recovery, and ventilatory efficiency, outside of heart failure-exclusive populations. Additionally, while resting echocardiographic data may provide some predictive value in assessing exercise capacity, CPET is still an important adjunct diagnostic tool for accurate evaluations of cardiorespiratory fitness that cannot be fully replaced by echocardiography. However, these results provide evidence of certain echocardiographic variables that may serve as potential indicators of reduced exercise capacity. Further studies are needed to evaluate various subpopulations, as well as age interaction effects with these echocardiographic variables.

## Figures and Tables

**Table 1:** Summary of inclusion and exclusion criteria.

Inclusion Criteria
- Age ≥ 18 years
- Underwent both a resting transthoracic echocardiogram and a maximum effort CPET (≥80% predicted VO2 max) within 3 months of each other
- CPET and echocardiogram performed between 1 January 2017 and 31 December 2022
**Exclusion Criteria**
- History of coronary artery bypass grafting (CABG)
- Congenital heart disease
- History of valvular repair or replacement
- Severe valvular stenosis or regurgitation (aortic, mitral, pulmonic, tricuspid), per ASE criteria
- Presence of pacemaker
- Atrial fibrillation or atrial flutter
- Pulmonary hypertension (RV systolic pressure > 40 mmHg)
- Left ventricular ejection fraction < 50%

**Table 2:** Demographics.

Total	(*n* = 1909)
Gender, *n* (%)	
Female	830 (43.5%)
Male	1079 (56.5%)
Age, *n* (%)	
18–35	394 (21.7%)
36–50	400 (22.0%)
51–65	582 (32.1%)
66–80	404 (22.3%)
80+	35 (1.9%)
BMI Mean (SD)	28.7 (6.27)
BSA Mean (SD)	2.0 (0.27)

BMI, Body Mass Index; BSA, Body Surface Area (m^2^).

**Table 3:** Echo parameter vs. percentage of predicted peak VO2 Correlations.

Echo Parameter	Correlation Coefficient ^1^	*p*-Value
Significant ^2^		
LV Stroke Volume Index (Doppler)	0.284	<0.0001
LV Volume Indexed End-Diastolic 2D by MOD Biplane	0.149	<0.0001
Tissue Doppler-Derived MV Medial Annular a’ Velocity	0.142	<0.0001
MV Medial Annular E-to-e’ ratio	−0.117	<0.0001
MV E Wave Peak Velocity	−0.109	<0.0001
Tissue Doppler-derived TV Lateral Annular Systolic Velocity (S’)	0.106	<0.0001
LA Volume Indexed 2D by MOD Biplane	0.105	<0.0001
TV Regurgitation Peak Velocity	−0.098	0.0003
MV Lateral Annular E-to-e’ ratio	−0.094	0.0001
RV Systolic Pressure	−0.089	0.001
LV Mass Indexed 2D	−0.087	0.0003
Non-Significant ^2^		
Tissue Doppler-Derived MV Lateral Annular a’ Velocity	0.078	0.0028
Aortic Mid-Ascending Diameter 2D	0.072	0.0055
MV E-to-A Ratio Diastolic	−0.054	0.0227
LV Ejection Fraction by 2D MOD Biplane	0.05	0.1273
MV Medial Annular e’ Peak Velocity	0.039	0.0962
MV Lateral Annular e’ Peak velocity	0.031	0.2031
Reported/Calculated Ejection Fraction	−0.023	0.3113
MV A Wave Peak Velocity	−0.017	0.4614

^1^ Obtained from Spearman correlations. ^2^ Statistical significance set at *p* < 0.0025 after Bonferroni correction. LV, left ventricle; MOD, Simpsons Method of Discs; MV, mitral valve; TV, tricuspid valve; LA, left atrial.

**Table 4:** Multiple linear regression for % predicted peak VO2 from echo parameters.

Variable	Coefficient	Standard Error	t-Value	*p*-Value
Significant ^1^				
LV Mass Index	−0.25669	0.05315	−4.83	<0.0001
LV Stroke Volume Indexed by Q Doppler	0.77772	0.12697	6.13	<0.0001
MV E wave Peak Velocity	−24.66488	6.59626	−3.74	0.0002
TV Regurgitation Peak Systolic Velocity	−12.38568	4.32824	−2.86	0.0044
Tissue Doppler-Derived TV Lateral Annular Systolic Velocity (S’)	119.46908	43.9882	2.72	0.0069
LA Volume Indexed 2D by MOD Biplane	0.30432	0.12732	2.39	0.0173
Nonsignificant ^1^				
LV Volume Indexed End-Diastolic 2D by MOD Biplane	0.13793	0.08419	1.64	0.1021
MV Medial Annular E-to-e’ ratio	0.38933	0.35125	1.11	0.2683
MV Medial Annular A Wave Peak Velocity	9.6412	42.3963	0.23	0.8202

^1^ Statistical significance set at *p* < 0.05; R^2^ = 0.191. LV, left ventricle; MOD, Simpsons Method of Discs; MV, mitral valve; TV, tricuspid valve; LA, left atrial.

**Table 5:** Mitral valve echo parameter correlation coefficients with heart rate recovery and the VE/VCO2 slope.

Mitral Valve Echo Parameter	HRR Coefficient	*p*-Value	VE/VCO2 Slope Coefficient	*p*-Value
A Wave Peak Velocity	−0.275	<0.0001	0.172	<0.0001
Lateral Annular e’ Peak Velocity	0.269	<0.0001	−0.27	<0.0001
Lateral Annular E-to-e’ Ratio	−0.252	<0.0001	0.227	<0.0001
E-to-A Ratio Diastolic	0.25	<0.0001	−0.174	<0.0001
Medial Annular e’ Peak Velocity	0.245	<0.0001	−0.284	<0.0001
Medial Annular E-to-e’ Ratio	−0.236	<0.0001	0.241	<0.0001

## Data Availability

The data supporting the findings of this study are not publicly available due to privacy and ethical restrictions. The data may be available from the corresponding author upon reasonable request and with appropriate institutional approval.

## Supplementary Materials

The following supporting information can be downloaded at: https://www.mdpi.com/article/10.3390/jcm14093013/s1, Table S1: Descriptive statistics of individual echo parameters; Figure S1: CPET Treadmill Protocols

## Abbreviations

The following abbreviations are used in this manuscript:

VO2	Volume of oxygen consumption
CPET	Cardiopulmonary exercise test
HRR	Heart rate recovery
VE/VCO2	Minute ventilation/volume carbon of dioxide expired
VIF	Variance inflation factor
CABG	Coronary artery bypass grafting
BMI	Body Mass Index
BSA	Body Surface Area
LV	Left ventricle
PWD	Pulsed wave Doppler
MOD	Simpson’s Method of Discs
CWD	Continuous wave Doppler
MV	Mitral valve
TV	Tricuspid valve
LA	Left atrial
RA	Right atrial

## References

[1] Clausen J.S.R., Marott J.L., Holtermann A., Gyntelberg F., Jensen M.T. (2018). Midlife Cardiorespiratory Fitness and the Long-Term Risk of Mortality. J. Am. Coll. Cardiol..

[2] Harber M.P., Kaminsky L.A., Arena R., Blair S.N., Franklin B.A., Myers J., Ross R. (2017). Impact of Cardiorespiratory Fitness on All-Cause and Disease-Specific Mortality: Advances Since 2009. Prog. Cardiovasc. Dis..

[3] Kunutsor S.K., Kurl S., Khan H., Zaccardi F., Rauramaa R., Laukkanen J.A. (2017). Oxygen uptake at aerobic threshold is inversely associated with fatal cardiovascular and all-cause mortality events. Ann. Med..

[4] Choi J., Park J.S., Choi H.J., Choi H.M., Hwang I.C., Yoon Y.E., Cho G.Y. (2023). Peak VO_2_ and VE/VCO_2_ exhibit differential prognostic capacity for predicting cardiac events. Eur. Heart J..

[5] Bassett D.R., Howley E.T. (2000). Limiting factors for maximum oxygen uptake and determinants of endurance performance. Med. Sci. Sports Exerc..

[6] Saltin B., Calbet J.A.L. (2006). Point: In health and in a normoxic environment, VO_2_ max is limited primarily by cardiac output and locomotor muscle blood flow. J. Appl. Physiol..

[7] Pearlman A.S., Ryan T., Picard M.H., Douglas P.S. (2007). Evolving trends in the use of echocardiography: A study of Medicare beneficiaries. J. Am. Coll. Cardiol..

[8] Sljivic A., Kleut M.P., Bukumiric Z., Celic V. (2018). Association between right ventricle two- and three-dimensional echocardiography and exercise capacity in patients with reduced left ventricular ejection fraction. PLoS ONE.

[9] Guazzi M., Myers J., Peberdy M.A., Bensimhon D., Chase P., Arena R. (2010). Cardiopulmonary exercise testing variables reflect the degree of diastolic dysfunction in patients with heart failure-normal ejection fraction. J. Cardiopulm. Rehabil. Prev..

[10] Tucker W.J., Lijauco C.C., Hearon C.M., Angadi S.S., Nelson M.D., Sarma S., Nanayakkara S., La Gerche A., Haykowsky M.J. (2018). Mechanisms of the Improvement in Peak VO_2_ With Exercise Training in Heart Failure with Reduced or Preserved Ejection Fraction. Heart Lung Circ..

[11] Pucci G., Alessio S., Russo A., Cerasari A., Dominioni I., Sanesi L., Filippucci L., Vaudo G. (2020). Relationship between echocardiographic and functional parameters in patients with heart failure undergoing cardiopulmonary exercise test. Minerva Cardioangiol..

[12] Ojima S., Kubozono T., Kawasoe S., Kawabata T., Salim A.A., Ikeda Y., Ohishi M. (2022). Peak oxygen uptake in cardiopulmonary exercise testing was associated with left ventricular diastolic dysfunction in patients with preserved ejection fraction. Eur. Heart J..

[13] Szijarto A., Tokodi M., Fabian A., Lakatos B.K., Shiida K., Tolvaj M., Eles Z., Magyar B., Soos A., Sydo N. (2023). Deep-learning based prediction of peak oxygen uptake in athletes using 2D echocardiographic videos. Eur. Heart J. Cardiovasc. Imaging.

[14] Erevik C., Kleiven Ø., Froysa V., Bjorkavoll-Bergseth M., Hansen M., Chivulescu M., Klaebo L., Dejgaard L., Skadberg Ø., Melberg T. (2022). Novel echocardiographic measures of myocardial work predicts physical performance during prolonged strenuous exercise. Eur. J. Prev. Cardiol..

[15] Kandels J., Stöbe S., Kogel A., Hepp P., Riepenhof H., Droste J.N., Stoeggl T., Marshall R.P., Rudolph U., Laufs U. (2023). Effect of maximum exercise on left ventricular deformation and its correlation with cardiopulmonary exercise capacity in competitive athletes. Echo Res. Pract..

[16] Kneffel Z., Horváth P., Petrekanits M., Németh H., Sidó Z., Pavlik G. (2007). Relationship between Relative Aerobic Power and Echocardiographic Characteristics in Male Athletes. Echocardiography.

[17] Arena R., Guazzi M., Myers J., Peberdy M.A. (2006). Prognostic value of heart rate recovery in patients with heart failure. Am. Heart J..

[18] Nissinen S.I., Mäkikallio T.H., Seppänen T., Tapanainen J.M., Salo M., Tulppo M.P., Huikuri H.V. (2003). Heart rate recovery after exercise as a predictor of mortality among survivors of acute myocardial infarction. Am. J. Cardiol..

[19] Aijaz B., Squires R.W., Thomas R.J., Johnson B.D., Allison T.G. (2009). Predictive value of heart rate recovery and peak oxygen consumption for long-term mortality in patients with coronary heart disease. Am. J. Cardiol..

[20] Gong J., Castro R.R., Caron J.P., Bay C.P., Hainer J., Opotowsky A.R., Mehra M.R., Maron B.A., Di Carli M.F., Groarke J.D. (2022). Usefulness of ventilatory inefficiency in predicting prognosis across the heart failure spectrum. ESC Heart Fail..

[21] Nayor M., Xanthakis V., Tanguay M., Blodgett J.B., Shah R.V., Schoenike M., Sbarbaro J., Farrell R., Malhotra R., Houstis N.E. (2020). Clinical and Hemodynamic Associations and Prognostic Implications of Ventilatory Efficiency in Patients with Preserved Left Ventricular Systolic Function. Circ. Heart Fail..

[22] Tsurugaya H., Adachi H., Kurabayashi M., Ohshima S., Taniguchi K. (2006). Prognostic impact of ventilatory efficiency in heart disease patients with preserved exercise tolerance. Circ. J. Off. J. Jpn. Circ. Soc..

[23] Guazzi M., Myers J., Peberdy M.A., Bensimhon D., Chase P., Pinkstaff S., Arena R. (2010). Heart Rate Recovery and Tissue Doppler Echocardiography in Heart Failure. Clin. Cardiol..

[24] Mashayekhi B., Mohseni-Badalabadi R., Hosseinsabet A., Ahmadian T. (2023). Correlation between Heart rate recovery and Left Atrial phasic functions evaluated by 2D speckle-tracking Echocardiography after Acute Myocardial infarction. BMC Cardiovasc. Disord..

[25] Ojima S., Kubozono T., Kawasoe S., Kawabata T., Salim A.A., Ikeda Y., Ohishi M. (2023). VE/VCO_2_ slope in cardiopulmonary exercise testing was associated with left ventricular diastolic dysfunction in patients with reduced ejection fraction. Eur. Heart J..

[26] Lewis G.D., Shah R.V., Pappagianopolas P.P., Systrom D.M., Semigran M.J. (2008). Determinants of Ventilatory Efficiency in Heart Failure. Circ. Heart Fail..

[27] Lang R.M., Badano L.P., Mor-Avi V., Afilalo J., Armstrong A., Ernande L., Flachskampf F.A., Foster E., Goldstein S.A., Kuznetsova T. (2015). Recommendations for cardiac chamber quantification by echocardiography in adults: An update from the American Society of Echocardiography and the European Association of Cardiovascular Imaging. J. Am. Soc. Echocardiogr..

[28] Nagueh S.F., Smiseth O.A., Appleton C.P., Byrd BF3rd Dokainish H., Edvardsen T., Flachskampf F.A., Gillebert T.C., Klein A.L., Lancellotti P., Marino P. (2016). Recommendations for the Evaluation of Left Ventricular Diastolic Function by Echocardiography: An Update from the American Society of Echocardiography and the European Association of Cardiovascular Imaging. J. Am. Soc. Echocardiogr..

[29] Vittinghoff E., Glidden D.V., Shiboski S.C., McCulloch C.E. (2005). Regression Methods in Biostatistics: Linear, Logistic, Survival, and Repeated Measures Models.

[30] Vella C.A., Robergs R.A. (2005). A review of the stroke volume response to upright exercise in healthy subjects. Br. J. Sports Med..

[31] Morganroth J., Maron B.J., Henry W.L., Epstein S.E. (1975). Comparative Left Ventricular Dimensions in Trained Athletes. Ann. Intern. Med..

[32] Hutchinson P.L., Cureton K.J., Outz H., Wilson G. (2008). Relationship of Cardiac Size to Maximal Oxygen Uptake and Body Size in Men and Women. Int. J. Sports Med..

[33] Osborne G., Wolfe L.A., Burggraf G.W., Norman R. (2008). Relationships between Cardiac Dimensions, Anthropometric Characteristics and Maximal Aerobic Power (VO_2_max) in Young Men. Int. J. Sports Med..

[34] Mahmod M., Pal N., Rayner J., Holloway C., Raman B., Dass S., Levelt E., Ariga R., Ferreira V., Banerjee R. (2018). The interplay between metabolic alterations, diastolic strain rate and exercise capacity in mild heart failure with preserved ejection fraction: A cardiovascular magnetic resonance study. J. Cardiovasc. Magn. Reson..

[35] Vanoverschelde J.J., Essamri B., Vanbutsele R., D’Hondt A., Cosyns J.R., Detry J.R., Melin J.A. (1993). Contribution of left ventricular diastolic function to exercise capacity in normal subjects. J. Appl. Physiol..

[36] Carrick-Ranson G., Hastings J.L., Bhella P.S., Shibata S., Fujimoto N., Palmer M.D., Boyd K., Levine B.D. (2012). Effect of healthy aging on left ventricular relaxation and diastolic suction. Am. J. Physiol. Heart Circ. Physiol..

[37] Zhao L., Zierath R., Claggett B., Dorbala P., Matsushita K., Kitzman D., Folsom A.R., Konety S., Mosley T., Skali H. (2023). Longitudinal Changes in Left Ventricular Diastolic Function in Late Life: The ARIC Study. JACC Cardiovasc. Imaging.

[38] Gardin J.M., Arnold A.M., Bild D.E., Smith V.E., Lima J.A., Klopfenstein H.S., Kitzman D.W. (1998). Left ventricular diastolic filling in the elderly: The cardiovascular health study. Am. J. Cardiol..

[39] Dalen H., Letnes J.M., Hoydal M.A., Wisløff U. (2024). Diastolic function and dysfunction in athletes. Eur. Heart J. Cardiovasc. Imaging.

[40] Hashimoto Y., Okamoto T. (2021). Arterial Stiffness and Left Ventricular Diastolic Function in Endurance Athletes. Int. J. Sports Med..

[41] Dalos D., Dachs T., Gatterer C., Schneider M., Binder T., Bonderman D., Hengstenberg C., Panzer S., Aschauer S. (2022). Cardiac remodeling in ambitious endurance-trained amateur athletes older than 50 years-an observational study. PLoS ONE.

[42] Teske A.J., Prakken N.H., De Boeck B.W., Velthuis B.K., Doevendans P.A., Cramer M.J. (2009). Effect of long term and intensive endurance training in athletes on the age related decline in left and right ventricular diastolic function as assessed by Doppler echocardiography. Am. J. Cardiol..

[43] Churchill T.W., Groezinger E., Kim J.H., Loomer G., Guseh J.S., Wasfy M.M., Isselbacher E.M., Lewis G.D., Weiner R.B., Schmied C. (2020). Association of Ascending Aortic Dilatation and Long-term Endurance Exercise Among Older Masters-Level Athletes. JAMA Cardiol..

[44] Gharacholou S.M., Scott C.G., Borlaug B.A., Kane G.C., McCully R.B., Oh J.K., Pellikka P.A. (2012). Relationship Between Diastolic Function and Heart Rate Recovery After Symptom-Limited Exercise. J. Card. Fail..

[45] Gardin J.M., Leifer E.S., Fleg J.L., Whellan D., Kokkinos P., LeBlanc M.-H., Wolfel E., Kitzman D.W. (2009). Relationship of Doppler-Echocardiographic left ventricular diastolic function to exercise performance in systolic heart failure: The HF-ACTION study. Am. Heart J..

[46] Fábián A., Lakatos B.K., Tokodi M., Kiss A.R., Sydó N., Csulak E., Kispál E., Babity M., Szűcs A., Kiss O. (2021). Geometrical remodeling of the mitral and tricuspid annuli in response to exercise training: A 3-D echocardiographic study in elite athletes. Am. J. Physiol. Heart Circ. Physiol..

[47] Studer Bruengger A.A., Kaufmann B.A., Buser M., Hoffmann M., Bader F., Bernheim A.M. (2014). Diastolic stress echocardiography in the young: A study in nonathletic and endurance-trained healthy subjects. J. Am. Soc. Echocardiogr..

[48] Hasegawa H., Little W.C., Ohno M., Brucks S., Morimoto A., Cheng H.J., Cheng C.P. (2003). Diastolic mitral annular velocity during the development of heart failure. J. Am. Coll. Cardiol..

[49] Zens T.J., Casar Berazaluce A.M., Jenkins T.M., Hardie W., Alsaied T., Tretter J.T., Moore R., Foster K., Fleck R.J., Hanke R.E. (2022). The Severity of Pectus Excavatum Defect Is Associated with Impaired Cardiopulmonary Function. Ann. Thorac. Surg..

