# Supplementary material for: Key Resting Echocardiographic Parameters for the Estimation of Exercise Parameters of Peak VO2, Heart Rate Recovery, and Ventilatory Efficiency
Source: J Clin Med. 2025 Apr 27;14(9):3013. doi: 10.3390/jcm14093013 (PMC12072650; doi:10.3390/jcm14093013)
Supplement: Supplementary file 1 [file jcm-14-03013-s001.zip › jcm-3572392-supplementary.pdf]

Supplemental Materials

1. Descriptive statistics of individual echo parameters

Echocardiographic Parameter	N (Missing)	Mean (SD)	Median (IQR)	Range
LV Mass Indexed 2D	1725 (184)	90.7 (32.86)	83.0 (70.0, 103.0)	35.0 – 309.0
MV E Wave Peak Velocity	1814 (95)	0.7 (0.18)	0.7 (0.6, 0.8)	0.3 – 1.8
MV A Wave Peak Velocity	1784 (125)	0.6 (0.23)	0.6 (0.4, 0.7)	0.1 – 5.0
MV E-to-A ratio Diastolic	1782 (127)	1.3 (0.60)	1.2 (0.9, 1.6)	0.1 – 5.0
MV Medial Annular e′ Peak Velocity	1798 (111)	0.1 (0.03)	0.1 (0.1, 0.1)	0.0 – 0.2
MV Medial Annular E-to-e' ratio	1774 (135)	9.3 (4.28)	8.3 (6.7, 10.0)	2.9 – 60.0
TV Regurgitation Peak Velocity	1392 (517)	2.3 (0.24)	2.3 (2.2, 2.5)	1.7 – 2.9
TV Regurgitation Mean Gradient	1392 (517)	22.1 (4.56)	22.0 (19.0, 25.0)	12.0 – 34.0
Right Atrial Pressure Estimate	1791 (118)	5.3 (1.39)	5.0 (5.0, 5.0)	3.0 – 20.0
Aortic Mid-Ascending Diameter 2D	1485 (424)	33.4 (5.15)	33.0 (30.0, 37.0)	21.0 – 52.0
LV Ejection Fraction by 2D MOD Biplane	944 (965)	61.1 (5.10)	62.0 (58.0, 64.0)	48.0 – 77.0
Tissue Doppler-derived MV Lateral Annular a′ Velocity	1464 (445)	0.1 (0.03)	0.1 (0.1, 0.1)	0.0 – 0.2
MV Lateral Annular e′ Peak velocity	1731 (178)	0.1 (0.04)	0.1 (0.1, 0.1)	0.0 – 0.2
Tissue Doppler-derived MV Medial Annular a′ Velocity	1506 (403)	0.1 (0.02)	0.1 (0.1, 0.1)	0.0 – 0.2
RV Systolic Pressure	1383 (526)	27.4 (4.72)	27.0 (24.0, 30.0)	16.0 – 39.0
MV Lateral Annular E-to-e' ratio	1706 (203)	7.0 (3.29)	6.3 (5.0, 8.2)	2.4 – 32.5
LV Stroke Volume Index (Doppler)	1726 (183)	45.0 (8.67)	44.0 (39.0, 50.0)	22.0 – 98.0
Tissue Doppler-Derived TV Lateral Annular Systolic Velocity (S′)	1632 (277)	0.1 (0.02)	0.1 (0.1, 0.2)	0.1 – 0.3

LA Volume Indexed 2D by MOD Biplane	1587 (322)	30.7 (10.68)	29.0 (24.0, 35.0)	10.0 – 98.0
LV Volume Indexed End-Diastolic 2D by MOD Biplane	940 (969)	67.1 (13.75)	67.0 (58.0, 75.0)	25.0 – 129.0
LV Stroke Volume Index (2D MOD Biplane)	927 (982)	40.7 (8.06)	40.0 (35.0, 46.0)	14.0 – 81.0
Reported/Calculated Ejection Fraction	1909 (0)	62.1 (5.38)	62.0 (59.0, 65.0)	50.0 – 80.0

2. CPET Treadmill Protocols

CARDIOPULMONARY TREADMILL PROTOCOLS

BRUCE PROTOCOL

THREE MINUTE STAGES

1.7 MPH	10 % GRADE
2.5 MPH	12 % GRADE
3.4 MPH	14 % GRADE
4.2 MPH	16 % GRADE
5.0 MPH	18 % GRADE
5.5 MPH	20 % GRADE
6.0 MPH	22 % GRADE
6.5 MPH	24 % GRADE

MODIFIED BRUCE

THREE MINUTE STAGES

1.7 MPH	0 % GRADE
1.7 MPH	5 % GRADE
1.7 MPH	10 % GRADE
2.5 MPH	12 % GRADE
3.4 MPH	14 % GRADE
4.2 MPH	16% GRADE
5.0 MPH	18 % GRADE
5.5 MPH	20 % GRADE
6.0 MPH	22 % GRADE

NAUGHTON PROTOCOL

TWO MINUTE STAGES

1.0 MPH	0 %	GRADE
2.0 MPH	0 %	GRADE
2.0 MPH	3.5 %	GRADE
2.0 MPH	7.0 %	GRADE
2.0 MPH	10.5 %	GRADE
2.0 MPH	14 %	GRADE
2.0 MPH	17.5 %	GRADE
3.0 MPH	12.5 %	GRADE
3.0 MPH	15 %	GRADE
3.0 MPH	17.5 %	GRADE
3.0 MPH	20. %	GRADE
3.0 MPH	22.5 %	GRADE
3.0 MPH	25 %	GRADE

MODIFIED NAUGHTON

TWO MINUTE STAGES

1.0 MPH	0 %	GRADE
1.5 MPH	3.5 %	GRADE
1.5 MPH	7.0 %	GRADE
1.5 MPH	10.5 %	GRADE
1.5 MPH	14 %	GRADE
1.5 MPH	17.5%	GRADE
1.5 MPH	24.5 %	GRADE
1.5 MPH	24.5 %	GRADE

MAYO PROTOCOL

TWO MINUTE STAGES

2.0 MPH	0 %	GRADE
2.0 MPH	7%	GRADE
2.0 MPH	14%	GRADE
3.0 MPH	12.5%	GRADE
3.0 MPH	17.5%	GRADE
3.4 MPH	18 %	GRADE
3.8 MPH	20 %	GRADE
5.0 MPH	18 %	GRADE
5.5 MPH	20 %	GRADE
6.0 MPH	22%	GRADE
6.5 MPH	24 %	GRADE

RUNNING PROTOCOLS

THREE MINUTE STAGE	2.5 MPH	0%
THREE MINUTE STAGE	5,6,7, 8 or 9 MPH	0%
TWO MINUTE STAGES	5,6,7,8 or 9 MPH	2.5%
	5,6,7,8 or 9 MPH	5.0%
	5,6,7,8 or 9 MPH	7.5%
	5,6,7,8 or 9 MPH	10.0%
	5,6,7,8 or 9 MPH	12.5%
	5,6,7,8 or 9 MPH	15.0%
	5,6,7,8 or 9 MPH	17.5%
